# CyberSentinel: A Transparent Defense Framework for Malware Detection in High-Stakes Operational Environments

**DOI:** 10.3390/s24113406

**Published:** 2024-05-25

**Authors:** Mainak Basak, Myung-Mook Han

**Affiliations:** School of Computing, Gachon University, Seongnam-si 13120, Republic of Korea

**Keywords:** explainable AI, malware classification, multi-branch network, attention block

## Abstract

Malware classification is a crucial step in defending against potential malware attacks. Despite the significance of a robust malware classifier, existing approaches reveal notable limitations in achieving high performance in malware classification. This study focuses on image-based malware detection, where malware binaries are transformed into visual representations to leverage image classification techniques. We propose a two-branch deep network designed to capture salient features from these malware images. The proposed network integrates faster asymmetric spatial attention to refine the extracted features of its backbone. Additionally, it incorporates an auxiliary feature branch to learn missing information about malware images. The feasibility of the proposed method has been thoroughly examined and compared with state-of-the-art deep learning-based classification methods. The experimental results demonstrate that the proposed method can surpass its counterparts across various evaluation metrics.

## 1. Introduction

Malware typically refers to malicious code designed to deface confidential data, financial information, or any digital resources within a computer system. Evaluating malicious software is an ongoing process [1,2]. The ease of communication, facilitated by the availability of the internet, digital assets, online transactions, and Internet of Things (IoT) devices, has accelerated the evolution of malware [3]. The impact of malware has reached alarming levels in recent years, with global damages estimated at $6 trillion in 2021, as per statistics [4]. Predictions suggest this cost will rise to $10 trillion by 2025. Despite these severe financial threats, understanding new malware variants to prevent catastrophes has become a complex task due to dynamic and heterogeneous computing infrastructures. Nevertheless, the cybersecurity research community is actively exploring innovative ideas to counter this unseen malware.

In the early days, malware was identified using signature-based approaches. These static detection methods scanned and compared suspicious files with predefined malware signatures heuristically [4,5,6,7]. It is worth noting that scanning-based malware defenders require a substantial number of handcrafted malware feature samples (such as text signs, regular expressions, filenames, byte codes, etc.) [1]. Despite feature engineering, these methods can only detect a limited number of malwares that remain unchanged from the predetermined features. Anti-analysis techniques such as obfuscation, packing, and polymorphism can easily evade these defenders with marginal modifications. Moreover, traditional approaches are computationally expensive as they necessitate setting up a secure environment before analyzing each suspicious file. These shortcomings highlight the impracticality of traditional handcrafted methods for dynamic malware classification. 

**Figure 1:**
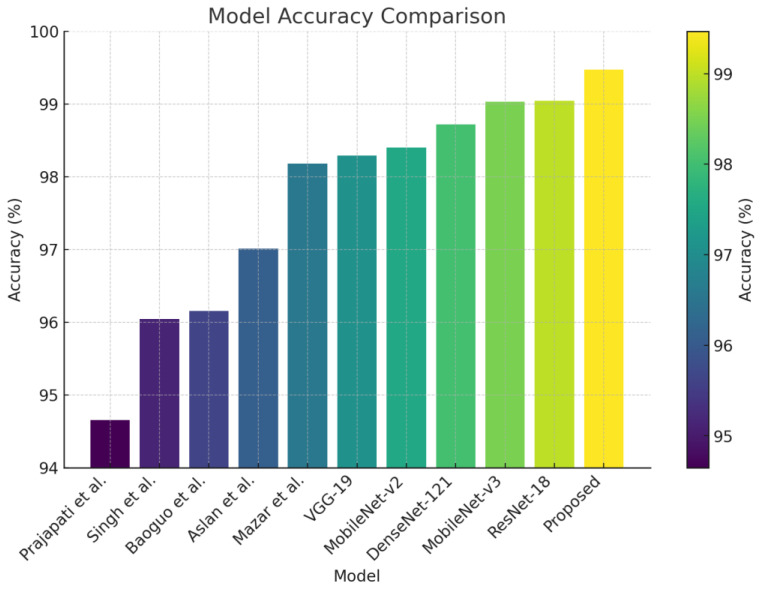
Comparison between different classification methods [5,6,11,12,13,14,15,16,17] with our method for malware classification.

Recent work on malware detection emphasizes developing Artificial Intelligence (AI)-driven solutions using deep learning to address the limitations of traditional malware detection approaches [1,2]. These learning-based methods treat malware analysis as a classical image classification task, representing malware binaries in image form. Over the past decade, several novel works with complex network architectures have been introduced [5,6,8,9,10], demonstrating significant improvement over traditional counterparts. To assess the practicality of existing methods for building a robust malware detection system, we evaluated the performance of various classification methods (including dedicated malware classification methods) on a benchmark malware dataset, as depicted in Figure 1.

As depicted in Figure 1, the current deep networks, specifically those employed in malware classification methods, fall short of achieving a high-fidelity rate in the benchmark dataset. Initial evaluations revealed that existing malware classification methods struggle to extract and utilize essential features from malware images. This limitation served as the driving force behind the development of a robust malware classifier intended for widespread application in cybersecurity scenarios. 

To address the limitations of existing methods, this study proposes a novel deep network designed for classifying malware classes. Our proposed deep network employs a two-branch structure to incorporate local-global attention across different image scales. One branch of the network integrates a DenseNet backbone [11] and a computationally efficient module inspired by spatial asymmetric attention [18] to extract and refine malware features from high-resolution images. The second branch (referred to as the auxiliary branch in subsequent sections) focuses on learning salient feature extraction from low-resolution malware images. This enables it to handle missing information in malware inputs arising from compression artifacts [19], attenuation, parsing errors, etc. We conducted a comprehensive evaluation of our proposed method, comparing it with existing malware classification methods. Our approach outperforms current deep malware methods by 1.25 in F1-score and 2.44 in precision. Additionally, this study highlights the feasibility of utilizing state-of-the-art (SOTA) deep classification models for future investigations.

The primary contributions of this study are as follows:

- We propose a novel deep network featuring attention and an auxiliary branch to capture salient features from malware images.
- Our proposed network integrates a faster asymmetric spatial-attention module (ASAM) with 65% lower computation efficiency, along with a dedicated auxiliary branch to leverage low-resolution inputs.
- A hyperparameter optimization algorithm was proposed based on Quantum Swarm Algorithm, referred to as QSOH. This optimization technique was utilized to overcome the disadvantage of traditional hyperparameter optimization methods, which approach local optima quickly.
- Our approach significantly outperforms the existing works on the benchmark dataset across multiple evaluation metrics.

The remainder of the paper is structured as follows: Section 2 reviews related works, Section 3 provides a detailed explanation of our proposed method, and Section 4 conducts a comprehensive evaluation and summarizes the results. Section 5 concludes the work.

## 2. Related Study

Machine learning-based malware analysis is a relatively recent addition to the cybersecurity domain. Based on the feature extraction technique, learning-based approaches can be categorized into two subgroups. This section provides a brief overview of both categories in the learning-based malware classification approach. Furthermore, it includes some recent literature on image-based malware detection schemes.

Traditional learning-based approaches for malware analysis heavily rely on manual feature extraction. Typically, these methods extract malware features manually and then employ these handcrafted features to feed shallow classifiers such as SVM, naive Bayes classifier, decision trees, k-nearest algorithms, etc. [4,14,16,17]. However, the performance of these solutions depends entirely on the quality of feature engineering. Shallow classification methods are also infamous for their scalability limitations [1]. In most realistic scenarios where the classifier must handle the task of categorizing an exponentially growing number of malware samples, these methods often fall short of achieving state-of-the-art performance. Therefore, recent works on malware classification have increasingly favored deep learning-based solutions.

Recent research applied deep learning like CNNs and RNNs, which can automatically learn relevant features from data, as shown by Alazab et al. [18]. Notably, Nataraj et al. [17] demonstrated using deep learning on images rendered from malware binaries to accurately detect malware. However, Bai et al. and Moser et al. emphasized that malware detection remains an ongoing challenge requiring new techniques to counter evolving threats. Nataraj et al. [17] proposed visualizing malware as images and using deep learning for automatic malware classification, achieving high accuracy.

Deep learning-based malware classifiers have demonstrated a significant performance improvement over traditional methods in the past decade. In a recent study, Gilbert et al. [8] introduced a LeNet-like stacked convolutional neural network for classifying malware images, achieving a validation accuracy of 99.37% on a 9-class malware dataset. Luo et al. [15] employed a local binary pattern (LBP) to extract malware features and utilized a similar network architecture as Gilbert et al. [8] on the Malimg dataset, reporting a classification accuracy of 93.17% on validation data. Aiden et al. [9] adopted a similar feature extraction architecture to their prior methods but replaced the softmax classifier with a support vector machine (SVM) classifier, achieving an accuracy of 77.23% on the Malimg dataset. However, on their setup, GRU-SVM outperformed their CNN-SVM structures by a notable margin. Ajay et al. [20] proposed a CNN with four consecutive blocks combining convolution and max-pooling operations, reaching 96.10% accuracy on the Malimg dataset. Yeo et al. [15] used a CNN with flow data to achieve just over 85% classification on a 9-class dataset. Kalash et al. [12] proposed a deep network called M-CNN, reporting an accuracy of 98.52% in the validation phase using the Malimg dataset. Yuan et al. [21] proposed a deep-stacked CNN with 13 convolution layers, achieving an accuracy of 99.26% on a 9-class malware dataset with a 10-fold validation strategy. Additionally, Prajapati et al. [19] compared different network architectures in their study with a 17-class malware dataset, reporting an accuracy of 89.55% with a 2D CNN architecture. They found that pre-trained Resnet-152 and VGG-19 architectures could outperform their 2D CNN with a marginal score. 

While various novel works explore different CNN architectures, it has been observed that training a CNN without pre-trained weights leads to unsatisfactory performance in malware classification. Notably, existing malware benchmark datasets lack data diversity, prompting recent works to utilize Imagenet pre-trained weights. For instance, Rezende et al. [22] proposed using a VGG-16 with Imagenet pre-trained weights, achieving a validation accuracy of 90.77% with a 10-fold validation strategy on a 20-class dataset. Similarly, Khan et al. [13] applied transfer learning on Resnet-18, 34, 50, 101, 152, and GoogleNet, achieving validation accuracies of 83%, 86.51%, 86.62%, 85.94%, 87.98%, and 84%. Mazhar et al. [6] leveraged a VGG-19 architecture with frozen weights and incorporated simple spatial attention, reporting an accuracy of 97.38% with class balancing on the Malimg dataset. Similarly, Aslan et al. [5] combined two pre-trained weights (AlexNet and Resnet0152) to classify malware images, reporting 97.18% accuracy on the Malimg dataset. 

Despite various novel approaches for classifying malware images, efforts in feature refinement are still insufficient. Arguably, without a focus on learning the salient features of malware inputs, achieving reliable and satisfactory performance in diverse malware classification may be challenging. This study aims to address the limitations of existing malware analysis works by proposing a novel deep method for learning salient features from malware images.

## 3. Materials and Methods

In this section, we outline the algorithm to parse malware binary op-code sequences into images. We further describe our proposed network architecture and our neural network model and provide all crucial background details required to comprehend the methodology being proposed.

The proposed malware classification framework is designed to optimize the detection and analysis of malicious files, incorporating a systematic methodology across two primary components: data preprocessing and feature extraction and classification. 

In the data preprocessing phase, raw malware data, predominantly in the form of Portable Executable (PE) files, are meticulously converted into a standardized format suitable for detailed analysis. This transformation process involves converting PE files into raw binary streams and subsequently normalizing these streams to a fixed width to facilitate uniform data representation across varying files. This step is critical as it preserves the inherent structural integrity of the data, which is crucial for the effective extraction of meaningful features. The whole process of the conversion is meticulously described in Algorithm 1.

Following the preprocessing stage, the framework advances to feature extraction and classification. This phase entails the application of analytical techniques aimed at identifying distinctive patterns and attributes within the malware samples. The extracted features are then utilized to train the proposed model. The classification component in the dual auxiliary branch employs an Asymmetric Spatial Attention Module (ASAM) block to categorically refine and differentiate the features between benign and malicious images based on the identified features. It further enhances the classification with the proposed Auxiliary Attention Block, to further classify malware binaries into their respective classes. This bifurcated approach not only significantly enhances the accuracy of malware detection but also streamlines the process, thereby improving both efficiency and scalability within cybersecurity measures.
**Algorithm 1** Conversion of PE malware binary File to 2D Image1:**Input:** PE file2:**Output:** 2D Image Matrix3:**Procedure:** PEtoImage(*PEfile*)4:      *binaryStream* ← ConvertToBinaryStream(*PEfile*)5:    *imageWidth* ← DetermineWidth(*binaryStream*)6:    *pixelValues* ← empty list7:    **for** each *byte* in *binaryStream*
**do**8:    *integerValue* ← BinaryToInteger(*byte*)9:    Append (*pixelValues*, *integerValue*)10:    **end for**
11:       *imageMatrix* ← ConvertTo2DMatrix(*pixelValues*, *imageWidth*)12:    *coloredImage* ← ApplyColorMap(*imageMatrix*)13:    **return** *coloredImage*
14:**end procedure**15:**function** ConvertToBinaryStream(*PEfile*)16:    *Read the PE file as a binary stream*
17:    **return** *binaryStream*
18:**end function**19:**function** DetermineWidth(*binaryStream*)20:    *Determine a fixed width based on the file size*
21:    **return** *width*
22:**end function**23:**function** BinaryToInteger(*byte*)24:    *Convert 8-bit binary substring to an unsigned integer*
25:    **return** *integerValue*
26:**end function**27:**function** ConvertTo2DMatrix(*pixelValues*, *width*)28:    *height* ←⌈len(*pixelValues*)*/width*⌉29:*Reshape the list of pixel values into a 2D matrix of dimensions height × width*30:    **Return** *matrix*
31:**end function**32:**Function** ApplyColorMap(*imageMatrix*)33:    *Apply an RGB color map to the 2D image matrix*
34:    **Return** *coloredImage*
35:**end function**

### 3.1. Data Preprocessing

**Figure 2:**
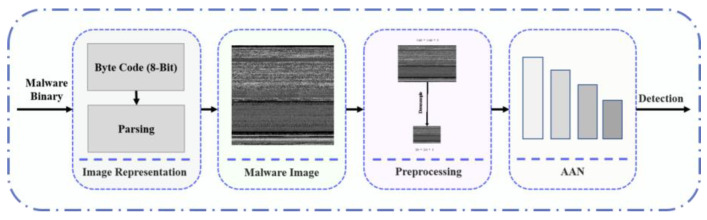
Overview of the proposed conversion method.

In the data preprocessing phase, raw malware data, predominantly in the form of Portable Executable (PE) files, are meticulously converted into a standardized format suitable for detailed analysis. This transformation process involves converting PE files into raw binary streams and subsequently normalizing these streams to a fixed width to facilitate uniform data representation across varying files. The dimensions of the images (length and width) are chosen based on the size of the binary stream to preserve the inherent structural integrity of the data, which is crucial for effective feature extraction. Figure 2 illustrates the overview of the proposed method of parsing malware Portable Executable (PE) files into 2D images. In this study, we process the malware binary input and represent it as 2D images. The process of parsing malware binary to image is shown in Algorithm 1.

In this investigation, an algorithm was devised to transform Portable Executable (PE) files into two-dimensional (2D) images, thereby enhancing the capabilities for malware detection through visual analysis. Initially, each PE file is converted into a raw binary stream, ensuring the preservation of essential structural information. A predetermined width, calculated based on the size of the binary stream, standardizes the image dimensions across different files. The binary stream is then segmented, and each segment is translated into pixel values, which are methodically organized into a 2D matrix. Subsequently, an RGB color map is applied to this matrix, facilitating the visual representation of the binary data. This method not only maintains the intrinsic structural patterns critical for identifying malicious content but also optimizes the data for subsequent image-based feature refinement for the proposed neural network. 

Subsequently, we rescale the image input into two different scales for feeding into the proposed network. Our network learns salient features from the provided malware image and classifies them based on the learned features. 

### 3.2. Proposed Model Architecture

The proposed architecture encapsulates a sophisticated deep learning framework designed to classify binary Portable Executable (PE) files into distinct malware families, leveraging techniques in image processing and neural networks. At its inception, the architecture tackles data preprocessing by transforming binary PE file content into 8-bit vector representations, which are then rendered as 2D grayscale images. This approach allows the model to utilize convolutional neural networks (CNNs), which excel in extracting patterns from image data. The preprocessing stage also includes data augmentation to enhance model robustness and generalizability by artificially expanding the training dataset with modified but realistic examples. This process involves various transformations, such as rotation, scaling, noise filter addition and interpolation, aimed at making the model more adept at handling different variations of input data.

**Figure 3:**
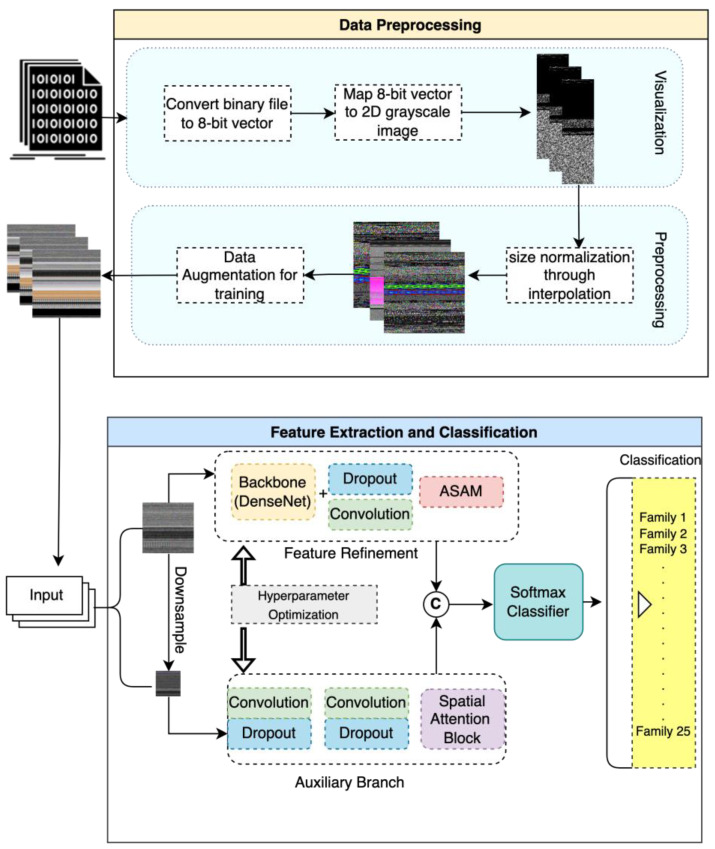
Overall proposed network architecture.

Subsequent to data preprocessing, the architecture advances to feature extraction and classification, structured meticulously (see Figure 3) to capture the most indicative features of malware images. The backbone of the feature extraction module is a DenseNet architecture, chosen for its efficacy in retaining important features through its dense connectivity pattern. This is followed by layers of convolution and strategic dropout layers to prevent overfitting, ensuring that the model generalizes well to new, unseen data. The inclusion of the proposed Asymmetric Spatial-Attention Mechanism (ASAM) further refines the features by emphasizing areas of the image that are more informative for classification. This feature refinement process is crucial for distinguishing between malware families that may exhibit subtle differences. Furthermore, we propose an addition auxiliary branch to handle the missing information that might arise due to obfuscation of the malware family. Finally, the processed features are fed into a softmax classifier that categorizes the input into one of several malware families, facilitating the detection and analysis of potential threats. This architecture not only highlights the integration of advanced deep learning techniques but also underscores a tailored approach to cybersecurity, aiming at high accuracy and robustness in malware classification.

**Figure 4:**
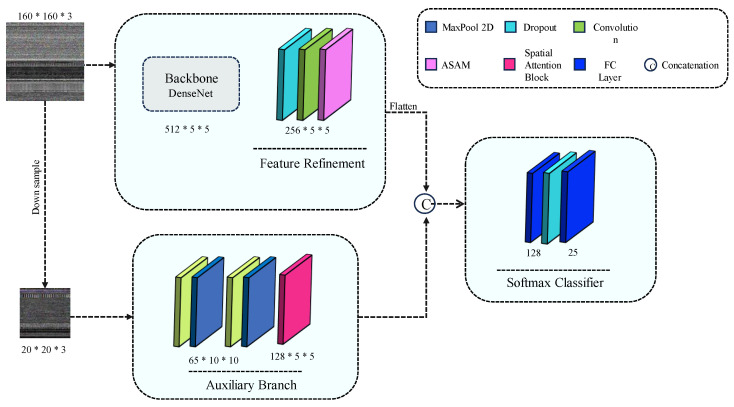
Proposed Auxiliary Attention Network (AAN).

The proposed Auxiliary Attention Network (AAN) consists of two distinct feature branches designed to learn salient features from different image scales. As depicted in Figure 4, our main branch takes an image input IM ∈ [0, 1]^H×W×3^, where H and W represent the height and width of the input. We incorporated a Densenet121 (without fully connected layers) [23] pre-trained block as the backbone to extract generic features. Subsequently, we introduced the ASAM mechanism to refine features extracted with local-global attention. Additionally, our proposed Auxiliary network includes a novel auxiliary attention branch, aiming to learn artifacts and missing spatial information that may arise due to compression [23] and binary-to-image conversion. We concatenated the refined features of the attention branches to feed into the softmax classifier. Furthermore, dropout layers were employed in our network to mitigate overfitting [23,24,25].

This section is further divided into subsections, which will entail a detailed description of the proposed methods and block structures of the proposed ASAM neural block, which is used in the network.

#### 3.2.1. Faster Asymmetric Spatial-Attention Module

**Figure 5:**
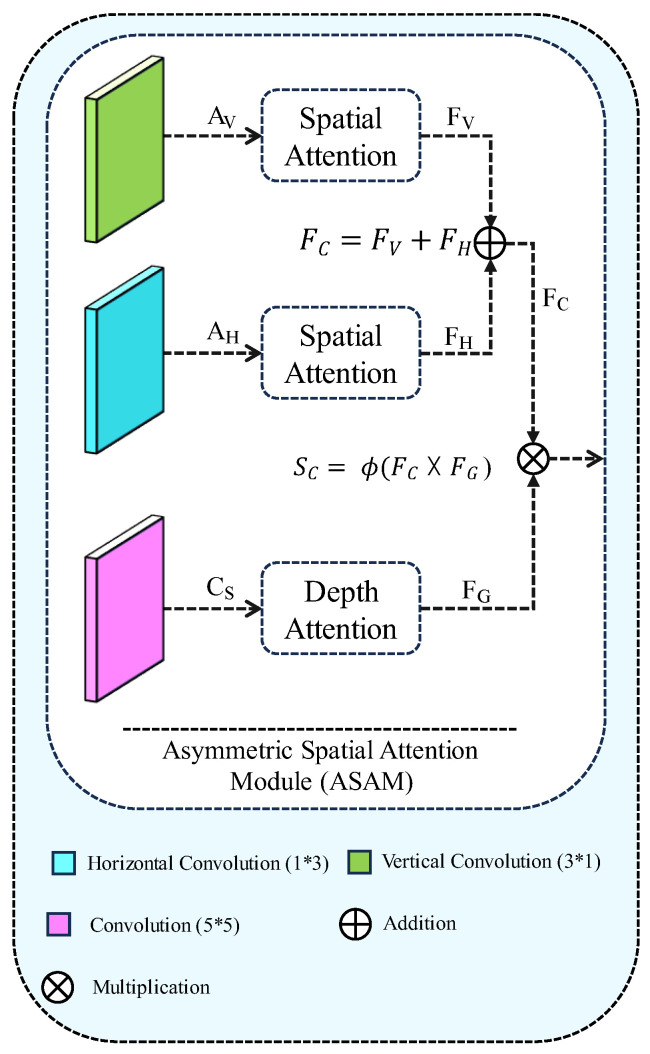
Proposed Asymmetric Spatial Attention Module (ASAM).

Asymmetric Attention Module (AAM) is renowned for its ability to refine features with global–local attention, demonstrating a significant impact in reconstructing non-Bayer images with real-world image noises. Despite its notable performance gain, AAM is computationally expensive, specifically employing a 9 × 9 convolution to achieve global attention from a given input. The process of Asymmetric Spatial Attention block is shown in Figure 5. 

However, malware classification faces substantial challenges due to data limitations. Consequently, utilizing such computationally expensive blocks for malware classification introduces two main issues: (i) a large number of trainable parameters leading to overfitting, and (ii) slower computation time.

To address both limitations, we propose replacing the large kernel convolution with a small kernel dilation convolution [26], as illustrated in Algorithm 2. This modification reduces the trainable parameters of the original AAM by 65% without compromising performance. Our approach to faster Spatial Attention Module (ASAM) is detailed as follows:

F_V_ = τ(C_S_([Z_A_(A_V_(X));Z_M_(A_V_(X))])) (1)

F_H_ = τ(C_S_([Z_A_(A_H_(X));Z_M_(A_H_(X))])) (2)

Here, A(·), C(·), and τ represent the asymmetric convolution operation, square convolution, and sigmoid activation, respectively. The symbol [;] denotes the concatenation of the two feature maps along the channel dimension. Additionally, Z_A_ and Z_M_ denote average pooling and max pooling to generate two 2D feature maps as X_A_ ∈ R^1×H×W^ and X_M_ ∈ R^1×H×W^. The mapped features are concatenated and presented as a 2D map.

In summary, the aggregated bidirectional attention over a given feature of malware is obtained as:

F_C_ = F_V_ + F_H_ (3)

**Algorithm 2** Asymmetric Spatial Attention Module1:**Procedure:** AsymmetricSpatialAttention2:**Input:** Feature map *X* ∈ *R^H^*^×*W*×*C*^3:**Output:** Enhanced feature map4:**Function** initialize (c_in_, c_out_, stride, padding mode)5:    Define convolutional layers for spatial processing:6:     *f_square_*(·) := Conv2d(·;kernel = (9,9),stride = *stride*,padding = 4)7:      *f_ver_*(·) := Conv2d(·;kernel = (3,1),stride = *stride*,padding = (1,0))8:      *f_hor_*(·) := Conv2d(·;kernel = (1,3),stride = *stride*,padding = (0,1))9:    Prepare activation and normalization functions10:**end function**
11:**function forward**(X)12:    Extract baseline features: *X_square_* = *f_square_*(*X*)13:    Apply directional convolutions:14:*V* = *f_ver_*(*X*),*H* = *f_hor_*(*X*)
15:Compute attention maps:
16:    *V_attn_* = *σ*(concat(*µ*(*V*),max(*V*)))17:     *H_attn_* = *σ*(concat(*µ*(*H*),max(*H*)))18:      *σ*(·): Sigmoid activation, *µ*(·): Mean pooling, max(·): Max pooling19:    Combine attention maps: *A* = *V_attn_* + *H_attn_*20:    Apply depth attention to enhance features:21:      *D* = DepthAttention(*X_square_*)22:    Integrate spatial and depth features: *Y* = *D* ⊙ *A*23:**return** *Y**▷* Return the final enhanced feature map24:**end function**


In the context, a squeeze-extractor block [10] has been harnessed as a global feature extractor utilized to pursue a spatial description as follows:

F_G_ = M_F_(Z_G_(C_D_(X))) (4)

**Figure 6:**
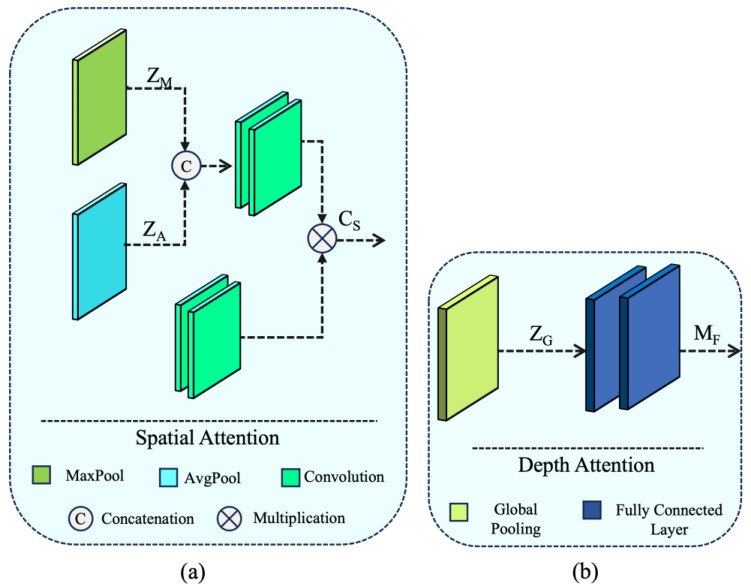
Overview of Proposed Attention blocks used Auxiliary branch (**a**) Spatial Attention block (**b**) Depth Attention block.

Here, M**_F_** and Z_G_ demote consecutive fully connected layers and global pooling operations (Figure 6b). It is worth noting that C_D_ in Equation (4) refers to the proposed small kernel dilated convolution operation. This operation enables us to reduce the trainable parameters of the proposed Faster ASAM by 65% compared with its base module.

#### 3.2.2. Auxiliary Attention Block

The auxiliary attention branch plays a crucial role in the proposed AAN, focusing on learning missing information resulting from compression artifacts, malware binary-to-image conversion, network effects, etc. The proposed auxiliary branch processes a low-sampled image, denoted as I_A_ ∈ [0,1]^H×W×3^, where H and W represent the height and width of the input. It applies two consecutive 3 × 3 convolutions and max-pooling operations before passing the extracted feature for refinement through a spatial attention block [27]. The spatial attention is implemented as follows:

F = τ(F_S_([Z_A_(X);Z_M_(X)]) (5)

Here, F(·) and τ represent the convolution operation and sigmoid activation, respectively. Additionally, Z**_A_** and Z**_M_** denote average pooling and max pooling, generating two 2D feature maps as X**_A_** ∈ R^1×H×W^ and X**_M_** ∈ R^1×H×W^. The spatial attention block [27] incorporated in the auxiliary branch is shown in Figure 6a.

Due to the polymorphic nature of malware binaries, malware op-code sequences, which include all the function call instructions for the executable files of the malware, may be obfuscated with redundant binaries of the malware signature. Therefore, when such binaries are parsed into images, certain sequences are hard to detect by the kernels of the neural network. Thus, to alleviate this shortcoming, the proposed auxiliary branch caters to extracting minute spatial details from the image sequence. The detailed logic flow is described in Algorithm 3.
**Algorithm 3 Auxiliary Attention Block (AAB)****Require:** Low-resolution image input *IA* ∈ [0,1]*^H^*^×*W*×3^**Ensure:** Refined feature map for classification **Input**: Down-sampled image *IA* from the primary network branch.2: **Output:** Refined features *F* for further classification. {Apply initial convolutional layers to extract low-level features} *IA*′ ← Apply two consecutive 3 × 3 convolutions to *IA*.4: *IA*″ ← Apply max pooling to *IA*′. {Spatial attention mechanism for feature refinement} *ZA*(*IA*″) ← Apply average pooling on *IA*″ to obtain the average feature map.6: *ZM*(*IA*″) ← Apply max pooling on *IA*″ to obtain the maximum feature map. {Concatenate pooled features and apply spatial attention} *F*′ ← *τ*(Conv([*ZA*(*IA*″)⊕*ZM*(*IA*″)])); where *τ* is the sigmoid function and Conv is a convolution operation.8: *F* ← Refine *F*′ through a spatial attention block to get the final attentionmodified feature map.**end process**

#### 3.2.3. Softmax Classifier

The softmax classifier of the proposed AAN processes the flattened and concatenated output from the feature branches. In this process, we applied a *tanh* activation to clamp the output of features from both branches before flattening. Subsequently, the concatenated features, with a dimension of 9600 vectors, were mapped into 512 using a fully connected layer. In the final layer, we utilized softmax activation with another fully connected layer to calculate the probability of respective malware classes.

### 3.3. Hyperparameter Optimization Based on Quantum Swarm Optimization

Adjusting hyperparameters has a significant impact on model performance, which is why the process of choosing and fine-tuning these parameters is a complex topic [28]. In convolutional neural networks, several key hyperparameters, such as layer count, neurons per layer, and learning rate, are crucial. Increasing the number of layers and neurons can significantly enhance the network’s feature extraction capabilities, which is beneficial for tackling more intricate problems. However, too many layers or neurons can reduce the generalization ability of the convolution networks [29]. The learning rate also plays a critical role in model convergence; a high learning rate can lead to rapid updates in backpropagation that may cause the loss function to oscillate and impede the model’s ability to converge [30]. On the other hand, a low learning rate might slow down the updating process too much, leading to slow convergence of the loss function and potentially causing the model to overfit.

In order to solve the issue of hyperparameter optimization, this study proposed a hyperparameter optimization algorithm based on quantum swarm optimization. This proposed novel method leverages the principles of quantum mechanics to enhance the exploration of the hyperparameter space beyond the capabilities of traditional algorithms. By employing a quantum behavior model, Quantum Swarm Optimization for Hyperparameters (QSOH) allows hyperparameters such as the number of network layers, neurons per layer, and learning rate to be optimized in a way that avoids local optima and accelerates convergence. This method addresses the issue of choosing an optimal set of hyperparameters without relying solely on empirical adjustments, which often lack a robust theoretical foundation and can lead to suboptimal performance (see Algorithm 4).
**Algorithm 4** QSHO Optimization1:**Input:***X*: Particle population representing hyperparameter sets2:**Output:** HParam best: the best Hyperparameter setting;3:Initialize particle population *X* with *velocity v*.4:**while** *t* < max iter do5:**for** Each Particle *i* in *X*
**do**6:P from [0,1].7:
  *X_i,new_* = *gbest* + *δ* × (*P* − 0.5)8:  where δ is the maximum step size, adjusted dynamically.9:  fitness*_i,new_* ← fitness function(*X_i,new_*)10:  **if** *fitness_i,new_* is better than *pbest_i_* **then**11:      *pbesti* ← *Xi,new*12:  **end if**
13:   **if** fitness*_i,new_* is better than *gbest* **then**14:      *gbest* ← *X_i,new_*15:  **end if**
16:  **end for**
17:  *t* ← *t* + 118:**end while**19:HP best ← *gbest* (Return the best global hyperparameter setting found)20:End Loop

In the algorithm, *GetBest* is the global optimization function for the particle population representing hyperparameter sets. *HParam*_*best* is the best hyperparameter setting. The formula for the update is shown below:

*X_i,new_* = *gbest* + δ × (*P* − 0.5) (6)

where *X_i,new_* is the updated fitness function of the new position, *gbest* is the global best setting on initial fitness and δ is the maximum step size and *P* is a uniform random number. 

This proposed algorithm leverages quantum mechanics principles to enhance the search capabilities and escape local optima effectively. By adjusting the positions using a probability amplitude influenced by the global best position, the QSOH method provides a robust and theoretically sound approach to optimizing hyperparameters in our proposed AAB network.

## 4. Experimental Results

This section illustrates the dataset descriptions, the results obtained through the proposed network, a comparison between different network architectures, the impact of hyperparameter variations on proposed network and an analysis of the results on high-stakes industry operational datasets. To evaluate our proposed model, we used the Malimg benchmark dataset to evaluate the performance of our proposed model. Furthermore, to evaluate our proposed model, we compared the performance result with the Microsoft Malware Classification Dataset [31] and BODMAS [32] dataset later in the section.

The performance of the proposed models is evaluated using the following metrics, i.e., *Accuracy*, *Precision*, *Recall* and F1-score.

(7) $$Accuracy=\frac{TP+TN}{TP+TN+FP+FN}$$

where *TP*, *TN*, *FP* and *FN* denote true positive, true negative, false positive and false negative, respectively.

Precision is calculated as:

(8) $$Precision=\frac{TP}{TP+FP}$$

Recall is calculated as:

(9) $$Recall=\frac{TP}{TP+FN}$$

F1-score is calculated as:

(10) $$\text{F1-score}=2\times \frac{precision\times recall}{precision+recall}$$

### 4.1. Dataset Preparation

To assess our proposed method, we utilized the Malimg benchmark malware image dataset [17] throughout this study, to train and evaluate the baseline model. The dataset comprises 9389 malware samples divided into 25 distinct classes, including well-known malware families such as Yuner.A, VB.AT, Malex.gen!J, Autorun.K, Rbot!gen, Swizzor.gen!I, C2Lop.p, etc. (shown in Table 1). Notably, these malware images are constructed from malware binaries. The binaries are converted to 8-bit vectors, from which we extracted the binary sequences and assigned pixel values to the 2D matrices.

#### 4.1.1. Details of Class Variants of the Microsoft Malware Classification Dataset

To evaluate the performance of our proposed model, we used Microsoft Malware Classification dataset [31]. In Table 2, a list of the samples is shown. It contains 9 malware classes from 10,868 malware binary files. The binary op-codes are parsed into vectorized images before feeding into the model. 

#### 4.1.2. Details of Class Variants of the BODMAS-14 Dataset

To further evaluate the model’s performance, we used BODMAS-14 [32] dataset (see Table 3). It consists of 14 classes and more than 1000 samples per class. The total number of binaries used and parsed was 32,389. The list of samples used is listed in Table 3.

**Figure 7:**
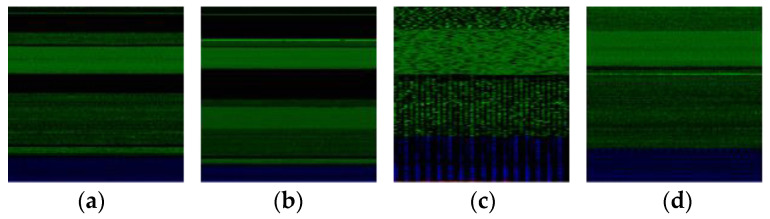
Malware images from Malimg dataset. (**a**) Adialer.C, (**b**) Autorun.K, (**c**) Wintrim.BX, and (**d**) Swizzor.geniE.

Figure 7 illustrates representative visualizations of malware images. We implemented a simple augmentation method by randomly flipping each image horizontally to mitigate overfitting during the training phase [23].

### 4.2. Implementation Details

The proposed method is implemented using the PyTorch framework [19]. We set the learning rate to 1 × 10^−4^ and adjusted it every two epochs during training, incorporating a weight decay of 1 × 10^−4^. The objective function of the proposed network is configured to minimize cross-entropy loss with an Adam optimizer. Additionally, we resized all training and testing images to 160 × 160 (for the main branch) and 20 × 20 for auxiliary branches during both training and testing. All models were trained for 30 epochs with a fixed batch size of 64. Our experiments were conducted on a machine equipped with an Intel (California, USA) i7-10700K @ 3.80 GHz × 16 central processing unit (CPU) clocked at 3.80 GHz, 16 GB of random-access memory, and an Nvidia GeForce GTX 1080Ti (16GB) graphical processing unit (GPU). 

### 4.3. Comparison

The proposed method has been compared with two genres of image classification methods: (i) Malware classification and (ii) SOTA image classification methods. To ensure a fair comparison, we trained all networks using our pre-processed dataset. Additionally, we re-trained and tested all models with images of the same dimensions (i.e., 160 × 160). Consequently, we adjusted the input layer of all comparison models to evaluate them based on our dataset. Subsequently, we summarized the performance of each deep model using standard evaluation metrics such as accuracy, F1 score, precision, and recall.

#### 4.3.1. Comparison with Malware Detection Methods

To compare and analyze existing malware classification models, we selected 11 state-of-the-art malware classification models that leverage deep learning. We trained each malware classification model with its suggested hyperparameters, allowing them to converge with the given dataset. Table 4 presents the performance of existing malware models on the benchmark dataset, considering our pre-processing and augmentation. The proposed methods consistently outperform existing methods across all evaluation metrics, achieving a performance gain of 1.25% in accuracy, 0.024 in precision, 0.0193 in recall, and a 0.0125 gain in F1-score. It is important to note that, unlike previous studies, we did not employ any weight balancing to enhance the scores. We evaluated all methods uniformly to simulate real-world scenarios. Despite the stringent evaluation strategy, the proposed method notably outperforms its counterparts by effectively learning salient features through two distinct feature branches.

#### 4.3.2. Comparison with Image Classification Methods on Malimg Dataset

Several deep network architectures have been introduced in the past decade for image classification, showcasing significant improvements and achieving high fidelity in classifying generic images. 

Several vision tasks have employed these networks as the backbone or directly adopted the network architecture to expedite their respective tasks. Recent works in malware classification, such as those by Aslan et al., Mazhar et al., etc., have also utilized SOTA image classification methods. While these studies have individually explored a few SOTA network architectures, there remains a need for an extensive evaluation of these image classification methods. This study fills this gap by thoroughly evaluating existing SOTA image classification models to understand their impact on malware classification.

To calculate the probability for 25 malware classes, we modified the final layer of the SOTA models and leveraged Imagenet pre-trained weights to achieve maximum performance. Table 5 illustrates the performance of SOTA image classification methods. As shown in Table 5, the proposed method significantly outperforms image classification methods in all evaluation metrics. It is noteworthy that several SOTA image classification methods, such as VGG-19, Squeezenet, MobileNet-v2, MobileNet-v3, Densenet121, etc., can outperform existing malware classification methods with a marginal score.

#### 4.3.3. Comparison with Existing Works with Proposed Method on Microsoft Malware Challenge Dataset

**Figure 8:**
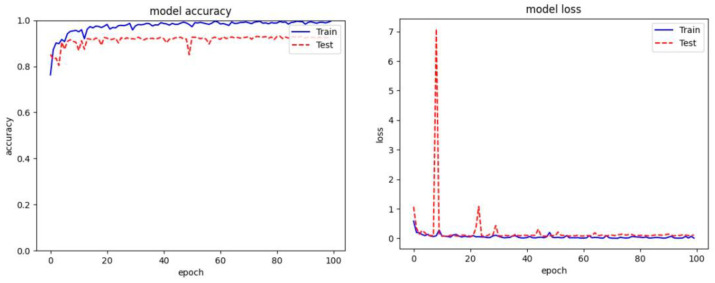
Evaluation graph of proposed model on Microsoft Malware dataset.

In our comprehensive analysis on the Microsoft Malware Challenge Dataset, our model demonstrated superior performance over existing state-of-the-art (SOTA) methods, notably achieving higher precision and recall metrics which contributed to an elevated F1-score (Table 6). This performance enhancement (see Figure 8) is attributable to our innovative integration of the faster Asymmetric Spatial-Attention Module (ASAM) and Auxiliary Attention Block (AAB), alongside the utilization of the Quantum Swarm Hyperparameter Optimization (QSHO) technique. These enhancements have proven particularly effective in discerning the nuanced patterns inherent in diverse malware types featured within the dataset. The success of our model not only underscores the efficacy of our methodological advancements but also highlights its potential applicability in real-world cybersecurity contexts, setting a new benchmark in malware detection capabilities.

#### 4.3.4. Comparison with Existing Works with Proposed Method on BODMAS-14 Dataset

**Figure 9:**
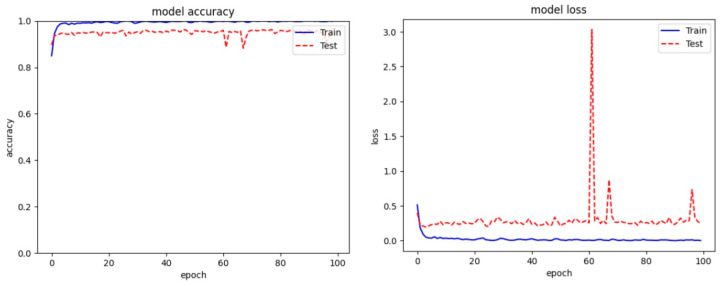
Evaluation graph of proposed model on BODMAS-14 dataset.

In our evaluation on the BODMAS-14 Dataset, our proposed model demonstrated superior performance compared to existing state-of-the-art models (Table 7). This dataset, characterized by its diverse and complex malware signatures, served as a rigorous test of our model’s adaptability and accuracy (see Figure 9). The effectiveness of our approach in this context highlights its advanced feature recognition capabilities, which were crucial in achieving high precision and robustness across various malware categories. This marked success underscores the practical relevance of our model and establishes it as a significant improvement over conventional methods in the field of malware detection, particularly in environments with varied and sophisticated threats.

### 4.4. Ablation Study

The malware detection system outlined demonstrates the capability to deliver superior outcomes, as illustrated in Table 8. Due to the model’s robust generalization abilities, it is expected to identify any new malware variants that share characteristics with the malware families already listed with similar levels of accuracy. This ensures that the system remains effective in recognizing and mitigating threats even as new malware emerges that is akin to known types.

The impact of each novel block was thoroughly studied through sophisticated experiments. In this analysis, we systematically removed proposed learning strategies, such as pre-trained weights, ASAM, auxiliary branch, etc., from the proposed network. Subsequently, each proposed module was individually injected to illustrate its impact on the final output. Table 9 demonstrates that the proposed modules have a meaningful impact on our reported final results. The ablation results further validate their feasibility in malware classification. 

**Figure 10:**
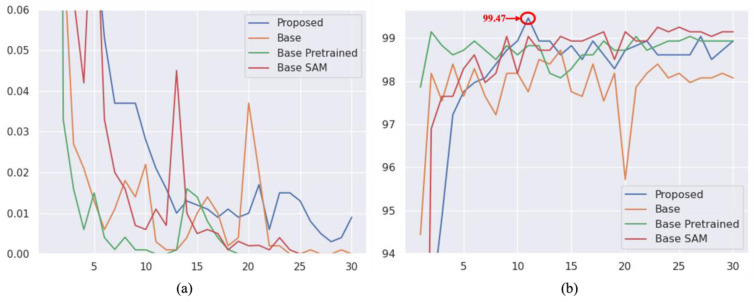
Overall Training result of proposed model on Malimg dataset. (**a**) Denotes model loss graph (**b**) Denotes model accuracy graph.

In addition to the objective scores, we visualized the validation accuracy and training loss during the training phase. As depicted in Figure 10, our proposed ASAM exhibits greater stability compared to its other variants of Spatial Attention Module (SAM). Moreover, the inclusion of dropout and our proposed Auxiliary Attention Block (AAB) noticeably aids our method in reducing overfitting and learning more useful features among the experimented variants.

### 4.5. Hyperparameter Optimization Variation Experiment

In order to verify the proposed QSOH algorithm, we compared the results with no QSOH, which includes without optimization, manual optimization and proposed optimization. This model was used on the Malimg benchmark dataset. To avoid interference from unbalanced data, we conducted the experiment on a fixed number of samples. The results of the study are shown in Table 10. 

To summarize the study, manual optimization obtained a better score than no optimization, without the proposed QSOH model achieving the highest performance result. This proves that for larger models, manual optimization fails to converge global optimum value. Moreover, such optimizations resolve the issue of a theoretical basis for hyperparameter optimization.

### 4.6. Classification Analysis

**Figure 11:**
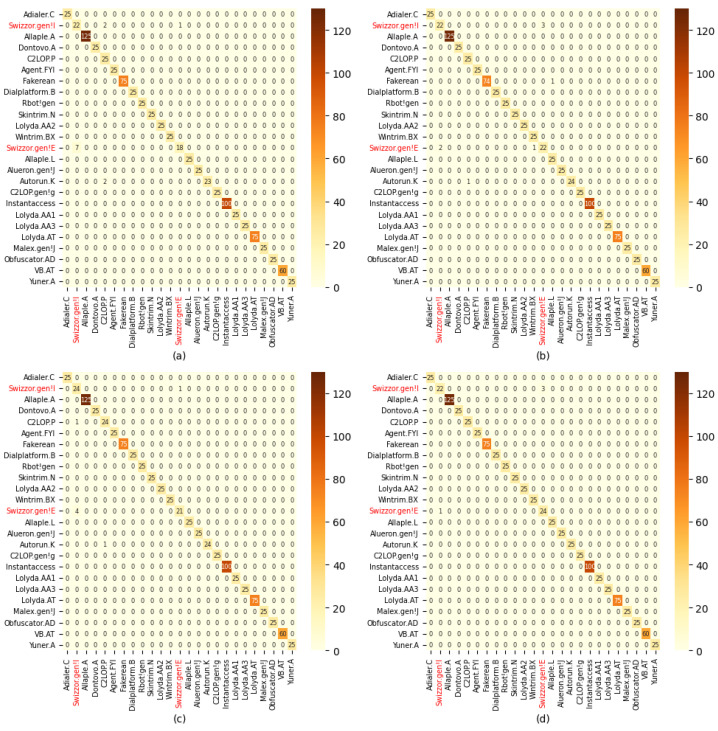
Confusion metrics of proposed AAN and its variants. (**a**) Base, (**b**) base pre-trained, (**c**) Base only ASAM (**d**) AAN (Proposed).

Figure 11 illustrates the class-wise malware predictions of the proposed AAN and its variants. It can be observed that malware from the same family (e.g., Swizzor.gen!I and Swizzor.gen!E) significantly impacts the performance of deep networks. Malware with homogeneous features is more challenging to identify. In this context, our proposed model demonstrates an ability to learn even such hard-to-distinguish features, enabling differentiation between closely related malware. Notably, our proposed Attention Block and Asymmetric Spatial-Attention Module (ASAM) contribute to the network’s capability to learn and refine salient information from malware images.

## 5. Discussion

The proposed method sheds light on various aspects of malware classification through sophisticated experiments. It highlights how efficient feature learning, coupled with a straightforward training strategy, can achieve state-of-the-art performance in malware classification. Despite facing severe data imbalance in the benchmark dataset, the proposed method outperforms existing methods without relying on any class information. Our Asymmetric Spatial-Attention Module (ASAM) and Auxiliary Attention Block (AAB) contribute to learning salient features for malware classification. The time-complexity details are mentioned in Table 11 below, which includes the inference of instances of floating-point operations and memory required for the model. From these values, it is evident that the model is lightweight and executable on edge devices.

While showcasing a significant improvement over existing works, the proposed method shares a common limitation with previous studies—lack of data diversity. Unfortunately, current benchmark datasets for malware classification exhibit noticeable limitations in terms of malware classes. As widely acknowledged, an increase in data samples within a dataset can substantially enhance the performance of deep learning models. Therefore, a meaningful future direction would involve collecting a diverse dataset with the maximum number of malware classes in a follow-up study.

The proposed method comprises 12.12 million trainable parameters, suggesting its potential applicability on edge devices. It would be intriguing to explore the performance of the proposed method on various edge devices with low computation resources in a future study, shedding light on its practical use in real-world applications.

## 6. Conclusions

This study proposes a two-branch deep network for malware classification. The proposed AAN includes a faster AAM to refine features extracted from a pre-trained densenet. Additionally, our model incorporates an auxiliary feature branch designed to learn salient missing features from low-quality malware images. The proposed network demonstrates significant performance improvement without employing any partial performance-enhancing techniques. We conducted a comprehensive comparison with various deep learning-based classification methods. In the future, we plan to extend the proposed study by deploying the model on edge devices. There are a few challenges remaining in the field of malware detection that will be the subject of our future study.

## Figures and Tables

**Table 1:** Details of Malimg dataset.

Family	ClassID	#varients
Allaple.L	0	22
Allaple.A	1	116
Yuner.A	2	2816
Lolyda.AA 1	3	1568
Lolyda.AA 2	4	193
Lolyda.AA 3	5	104
C2Lop.P	6	144
C2Lop.gen!g	7	166
Instantaccess	8	177
Swizzor.gen!I	9	162
Swizzor.gen!E	10	320
VB.AT	11	52
Fakerean	12	213
Alueron.gen!J	13	183
Malex.gen!J	14	122
Lolyda.AT	15	156
Adialer.C	16	17
Wintrim.BX	17	16
Dialplatform.B	18	148
Dontovo.A	19	80
Obfuscator.AD	20	126
Agent.FYI	21	132
Autorun.K	22	122
Rbot!gen	23	94
Skintrim.N	24	797

**Table 2:** Details of Microsoft Malware Classification dataset.

Family	ClassID	#varients
Rammit	0	1541
Lollipop	1	2478
Kelihos_ver3	2	2942
Vundo	3	475
Simda	4	42
Tracur	5	751
Kelihos_ver1	6	398
Obfuscator.ACY	7	1228
Gatak	8	1013

**Table 3:** Details of BODMAS dataset.

Family	ClassID	#varients
sfone	0	4729
wacatac	1	4694
upatre	2	3901
wabot	3	3673
small	4	3339
ganelp	5	2232
dinwod	6	2057
mira	7	1960
Berbew	8	1749
sillyp2yp	9	1616
ceeeinject	10	1169
gepys	11	1124
Benjamin	12	1071
musecador	13	1054

**Table 4:** Comparison with malware image classification methods on Malimg dataset.

Model	Accuracy	Precision	Recall	F1-Score
Ajay et al. [23]	96.04	0.9096	0.9239	0.9595
Aiden et al. [9]	94.55	0.8777	0.8957	0.9469
Yeo et al. [28]	93.47	0.8682	0.8851	0.9351
Luo et al. [15]	94.44	0.8926	0.9048	0.9438
Kalash et al. [12]	96.04	0.9151	0.9253	0.9608
Prajapati et al. [19]	94.65	0.899	0.9168	0.9459
Yuan et al. [28]	96.15	0.9256	0.9408	0.9618
Aslan et al. [5]	97.01	0.9341	0.9397	0.9708
Gibert et al. [8]	95.29	0.9075	0.92094	0.9528
Edmar et al. [21]	94.33	0.8719	0.8855	0.9448
Mazhar et al. [6]	98.18	0.9678	0.9724	0.9823
**AAN (Proposed)**	**99.47**	**0.9922**	**0.9915**	**0.9948**

**Table 5:** Comparison with state-of-the-art image classification models.

Model	Accuracy	Precision	Recall	F1-Score
VGG-16	97.86	0.9641	0.9614	0.9778
VGG-19	98.29	0.9713	0.9703	0.9833
AlexNet	95.08	0.8943	0.9116	0.9501
Densenet121	98.72	0.9806	0.9838	0.9875
Efficientnet	97.75	0.9528	0.9537	0.9775
GoogLenet	97.97	0.9629	0.9666	0.9802
Mobilenet-v2	98.40	0.9673	0.9607	0.9837
Mobilenet-v3	99.03	0.9773	0.9790	0.9906
Resnet18	99.037	0.9835	0.9819	0.9906
Shufflenet-v2	98.08	0.9637	0.9710	0.9806
Squeezenet1	98.29	0.9721	0.9712	0.982
Swin	97.86	0.9594	0.9609	0.97783
VIT-B-16	96.26	0.9273	0.9322	0.9622
Wide ResNet	95.94	0.935	0.9259	0.9598
**AAN (Proposed)**	**99.47**	**0.9922**	**0.9915**	**0.9948**

**Table 6:** Comparison with SOTA classification methods on Microsoft Malware dataset.

Model	Accuracy	Precision	Recall	F1-Score
Ajay et al. [23]	94.24	0.9423	0.9289	0.9356
Aiden et al. [9]	93.57	0.8777	0.8957	0.9469
Yeo et al. [28]	91.27	0.9221	0.9132	0.9176
Luo et al. [15]	80.51	0.8135	0.7986	0.8060
Kalash et al. [12]	93.4	0.9328	0.9354	0.9341
Prajapati et al. [19]	97.2	0.9761	0.9679	0.9720
Yuan et al. [28]	96.48	0.9646	0.9544	0.9595
Aslan et al. [5]	96.3	0.963	0.9582	0.9606
Gibert et al. [8]	93.72	0.9413	0.9254	0.9333
Edmar et al. [21]	96.08	0.9576	0.9616	0.9596
Mazhar et al. [6]	97.18	0.9657	0.9685	0.9671
**AAN (Proposed)**	**98.47**	**0.9828**	**0.9815**	**0.9848**

**Table 7:** Comparison with SOTA classification methods on BODMAS-14 dataset.

Model	Accuracy	Precision	Recall	F1-Score
Prajapati et al. [19]	97.2	0.9761	0.9679	0.9720
Luo et al. [15]	80.51	0.8135	0.7986	0.8060
Kalash et al. [12]	93.4	0.9328	0.9354	0.9341
Edmar et al. [21]	96.08	0.9576	0.9616	0.9596
Roseline et al. [33]	97.43	0.9753	0.9732	0.9742
Singh et al. [34]	96.08	0.9576	0.9616	0.9596
Yuan et al. [28]	96.48	0.9646	0.9544	0.9595
Aslan et al. [5]	96.3	0.963	0.9582	0.9606
Gibert et al. [8]	93.72	0.9413	0.9254	0.9333
Mazhar et al. [6]	97.18	0.9657	0.9685	0.9671
**AAN (Proposed)**	**98.24**	**0.9858**	**0.9764**	**0.9825**

**Table 8:** Comparison of performance with TOP malware classification datasets.

Metrics	Malimg Dataset	Microsoft Dataset	BODMAS-14 Dataset
Accuracy (%)	99.47	97.72	96.81
Precision	0.9922	0.9756	0.9650
Recall	0.9915	0.9748	0.9681
F1-score	0.9948	0.9752	0.9665

**Table 9:** Ablation study with different network variants of the proposed AAN.

Network Varient	PW	ASAM	AB	Param. (M)	Accuracy (%)	Precision	Recall	F1-Score
Base	X	X	X	8.227	98.72	0.9799	0.9810	0.9875
BasePre-trained	Y	X	X	8.22	99.14	0.9857	0.9865	0.9917
Base ASAM	Y	Y	X	10.26	99.25	0.9888	0.9869	0.9927
**(Proposed)**	Y	Y	Y	12.12	**99.47**	**0.9922**	**0.9915**	**0.9948**

**Table 10:** Results of hyperparameter optimization experiment.

Model	Accuracy	Precision	Recall	F1-Score
Without optimization	97.61	0.9720	0.9671	0.9788
Manual optimization	99.21	0.9913	0.9823	0.9932
QSHO **(Proposed)**	99.67	0.9956	0.99	0.9977

**Table 11:** Time-complexity inference.

Input	160 × 160 × 3
Auxiliary Input	20 × 20 × 3
Trainable Param (M)	12.12
Memory (MB)	20.32
Complexity Gmac	2.9
Inference Time (ms)	0.2

## Data Availability

Data is available from the corresponding author upon reasonable request.
